# Crystal Quality and Light Output Power of GaN-Based LEDs Grown on Concave Patterned Sapphire Substrate

**DOI:** 10.3390/ma8041993

**Published:** 2015-04-22

**Authors:** YewChung Sermon Wu, A. Panimaya Selvi Isabel, Jian-Hsuan Zheng, Bo-Wen Lin, Jhen-Hong Li, Chia-Chen Lin

**Affiliations:** Department of Materials Science and Engineering, National Chiao Tung University, 1001 University Road, Hsinchu 300, Taiwan; E-Mails: selvi.isabel@gmail.com (A.P.S.I.); one123.piece@msa.hinet.net (J.-H.Z.); lbwferro@gmail.com (B.-W.L.); lijhenhong.mse99@nctu.edu.tw (J.-H.L.); jdmhicp@yahoo.com.tw (C.-C.L.)

**Keywords:** GaN-based LED, concave patterned sapphire substrate, crystal quality, light output power

## Abstract

The crystal quality and light output power of GaN-based light-emitting diodes (LEDs) grown on concave patterned sapphire substrate (CPSS) were investigated. It was found that the crystal quality of GaN-based LEDs grown on CPSS improved with the decrease of the pattern space (percentage of c-plane). However, when the pattern space decreased to 0.41 μm (S0.41-GaN), the GaN crystallinity dropped. On the other hand, the light output power of GaN-based LEDs was increased with the decrease of the pattern space due to the change of the light extraction efficiency.

## 1. Introduction

High-brightness GaN-based light-emitting diode (LED) is widely used in variety of applications for their versatile functions like the display screens, mobile phones, automobile headlights, *etc.* The external quantum efficiency (EQE) is a function of both the internal quantum efficiency (IQE) and light extraction efficiency (LEE). While the GaN-based LEDs posses high luminescence efficiency they still suffer from poor epitaxial quality/IQE and poor LEE [1,2,3,4,5,6,7,8]. Patterned Sapphire substrate (PSS) technique can improve both the epitaxial quality and LEE [9,10,11,12,13].

In this study, micro-sized concave-PSS (CPSS) were fabricated with different pattern spaces. After the growth of the GaN layer, the crystallinity and performance of LEDs were compared and discussed.

## 2. Experimental

**Figure 1:**
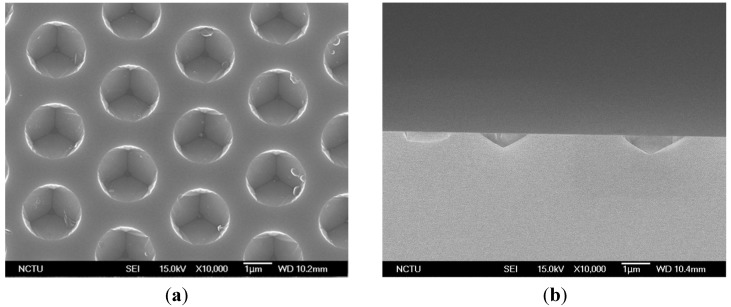
SEM images of (**a**) top-view; and (**b**) cross-section of S0.41 concave patterned sapphire substrate (CPSS).

Two-inch *c-*plane sapphire with 200-nm SiO_2_ hard mask was immersed in a mixture etchant of H_2_SO_4_:H_3_PO_4_ (3:1) at 270 °C. The SiO_2_ mask was then removed by a buffer-oxide etching (BOE) solution. The pattern pitch was 3 μm with different pattern spaces. Figure 1 shows scanning electron microscope (SEM) images of top view and cross-sectional view of CPSS. The diameter of the concave pattern was 2.59 μm and the space was 0.41 μm, which was denoted as S0.41. The related detail and percentage of c-plane were shown in Table 1. S0.60 and S0.87 CPSS were also used in this study.

**Table 1:** Diameter and spacing of CPSS with various percentage of c-plane.

Sample	Pattern size-Diameter/Spacing (µm)	Percentage of c-plane (%)
S0.41	2.59/0.41	32.40
S0.60	2.40/0.60	41.96
S0.87	2.13/0.87	54.28
S3.00(FLAT-GaN)	0.00/3.00	100

After the clean process, the LED structures were grown by metalorganic chemical vapor deposition (MOCVD). The structures consisted of a AlN as buffer layer on sapphire substrate, an undoped-GaN layer film, a n-GaN layer, a Si-doped AlGaN cladding layer, an InGaN-GaN multiple quantum wells (MQWs), a Mg-doped AlGaN cladding layer and a p-GaN layer. In this study the chip had an area of 10 × 23 mil^2^ (254 × 584 μm^2^). For the purpose of comparison, LED structures were also grown on flat sapphire without any pattern and denoted as FLAT-GaN/S3.00-GaN (~pattern space 3 μm, diameter 0 μm).

## 3. Results and Discussion

**Figure 2:**
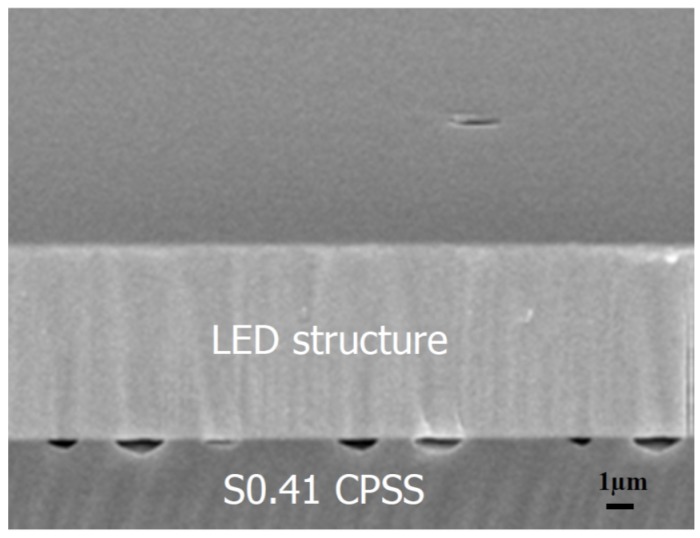
Cross-sectional SEM image of S0.41-GaN with voids between light-emitting diode (LED) and CPSS.

Figure 2 shows the cross-sectional SEM image of S0.41-GaN. Voids were found at the GaN/sapphire interface. The GaN crystal quality was analyzed by (1) X-ray diffraction (XRD); (2) photo luminescence (PL) and (3) screw dislocation density, which can be characterized by etching pit density (EPD).

The nature of GaN crystal qualities on sapphire substrates was first analyzed by XRD rocking curves. The full width at half maximum (FWHM) of (002) symmetric plane and the (102) asymmetric plane of GaN are tabulated in Table 2. A more or less continuous increase in GaN crystallinity (decrease in FWHM width) was expected with decreasing pattern space (percentage of c-plane). This is because most of the growth of GaN was initiated not from sidewall surfaces but c-planes [14,15,16,17]. As the growth time increased, GaN epilayers on the top c-plane covered these concave patterns by lateral growth causing the threading dislocation to bend. At the same time, voids were formed when growth front boundaries coalesced, as shown in Figure 2. The crystal quality was improved with the increase of lateral growth area of GaN. In other words, it improved with the decrease of percentage of c-plane (the pattern space). However, when the pattern space decreased to 0.41 μm, the crystallinity of S0.41-GaN dropped. We believe this drop is because further decrease in c-plane area makes epitaxy of GaN film on PSS very difficult [18].

**Table 2:** Characteristics of LEDs.

FWHM of XRCs (arcsec)			Forward Voltages (V)	LOP (mW)	Simulation of LEE (%)
	(002)	(102)	@ 20 mA	@ 20 mA	
S0.41-GaN	340.2	471.2	2.79	140.7	16.59
S0.60-GaN	336.6	442.8	2.79	115.5	16.07
S0.87-GaN	364.0	460.4	2.80	99.9	15.23
S3.00-GaN	369.7	502.6	–	–	9.19

**Figure 3:**
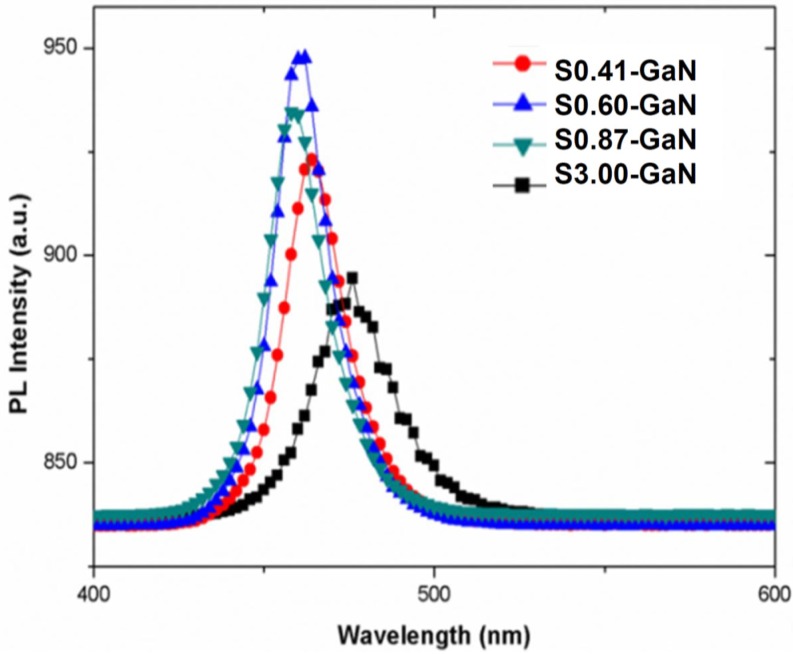
The photoluminescence of muti-quantum-wells grown on CPSS.

PL was also used to investigate the quality of muti-quantum wells (MQWs). The excitation source was a 325 nm, 30 mW He–Cd continuous wave laser. As shown in Figure 3, the peak intensity increased with the decrease of pattern space. Then, the intensity drop when pattern space decreased to 0.41 μm. These results also indicated that the crystallinity of S0.60-GaN was better than that of S0.87-GaN, S0.41-GaN and S3.00-GaN.

**Figure 4:**
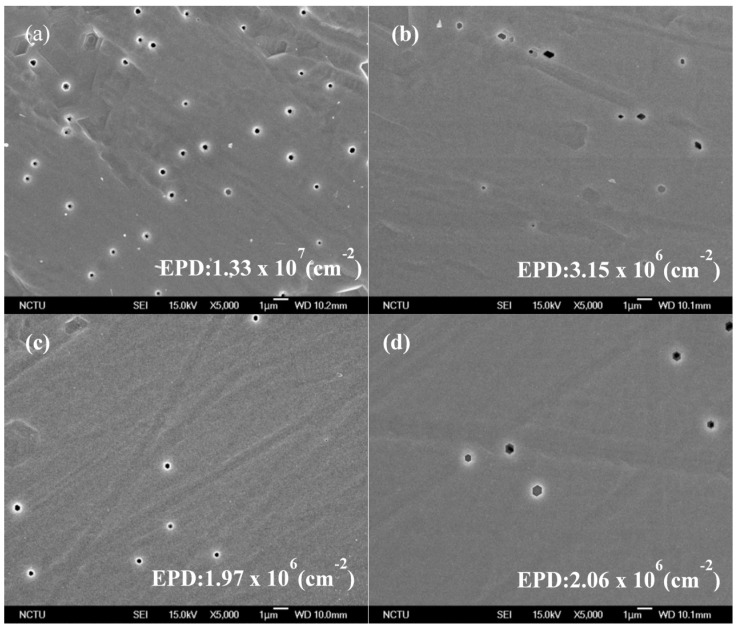
SEM images and Etching Pit density (EPD) of (**a**) S3.00-GaN; (**b**) S0.41-GaN; (**c**) S0.60-GaN and (**d**) S0.87-GaN.

Etching Pit density (EPD) was done by etching all four samples with H_3_PO_4_ etching solution at 210 °C for 5 min to investigate the GaN quality are shown in Figure 4. The measured EPD of S0.60-GaN was 1.97 × 10^6^ cm^−2^, which was less than that of S0.87-GaN (2.06 × 10^6^), S0.41-GaN (3.15 × 10^6^) and S3.00-GaN (1.33 × 10^7^). These results also confirm that the crystallinity of S0.60-GaN was better than that of S0.87-GaN, S0.41-GaN and S3.00-GaN.

The light output power characteristics of LEDs are listed in Table 2, the forward voltage of S0.41-GaN at 20 mA was 2.79 V, which was almost the same as that of S0.87-GaN and S0.60-GaN. However, surprisingly, the light output power (LOP) of S0.41-GaN at 20 mA was 140.7 mW, which was better than that of S0.60-GaN (115.5) and S0.87-GaN (99.9), even though the GaN crystal quality of S0.41-GaN was not as good as that of S0.60-GaN and S0.87-GaN. This observation suggested that the LEE of S0.41-GaN must be higher than that of S0.60-GaN.

**Figure 5:**
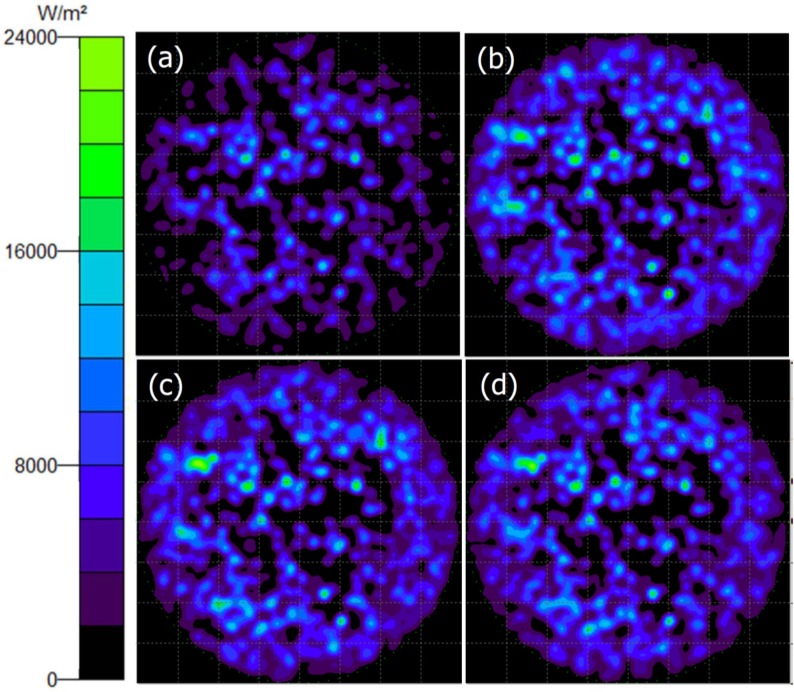
Simulation data of (**a**) S3.00-GaN; (**b**) S0.41-GaN; (**c**) S0.60-GaN and (**d**) S0.87-GaN.

The simulation data confirmed that the LEE of S0.41-GaN (16.59%) is higher than that of S0.60-GaN (16.07%) and S0.87-GaN (15.23%), as listed in Table 2. The simulation was performed using the Trace-Pro software, as shown in Figure 5. In this simulation, the thickness of sapphire, n-GaN, MQW and p-GaN were 400 μm, 2.5 μm, 58 nm and 100 nm, respectively; while their refractive indexes were 1.78, 2.42, 2.54 and 2.45, respectively [19]. Here, 200 mW power (5000 light rays) is assumed to emit randomly from the MQW.

Theoretical study indicates that EQE and LOP are a product of IQE and LEE. IQE is a function of crystal quality, while LEE is a function of interface/surface texture. In our study it was found that the crystallinity of S0.60-GaN was better than that of S0.87-GaN and S0.41-GaN. However, the LEE of S0.41-GaN was comparable and higher than other LEDs. As a result, the LOP (EQE) of S0.41-GaN was better than that of other LEDs.

## 4. Conclusions

In this study, CPSS was fabricated by wet-etching method. The pattern pitch was 3 μm with different pattern spaces. The crystal quality and light output power of GaN-based LEDs grown on CPSS were investigated. XRD rocking curves, PL and EPD analysis revealed that the GaN crystallinity was increased with the decrease of pattern space (percentage of c-plane). This is because most of the growth of GaN was initiated from c-planes. As the growth time increased, GaN epilayers on the c-plane covered these concave patterns by lateral growth causing the threading dislocation to bend. However, when the pattern space decreased to 0.41 μm, the crystallinity of S0.41-GaN dropped. This is because further decrease in c-plane area makes epitaxy of GaN film on PSS very difficult. The light output power of S0.41-GaN was better than others. This is because the LEE of S0.41-GaN was larger than other LEDs. 
